# Facilitators and barriers to the delivery of the PARAMEDIC2 trial

**DOI:** 10.1016/j.resplu.2024.100617

**Published:** 2024-03-27

**Authors:** Helen Pocock, Nigel Rees, Imogen Gunson, Mark Docherty, Karl Charlton, Michelle Jackson, Charlotte Scomparin, Ed England, Rachael Fothergill

**Affiliations:** a South Central Ambulance Service NHS Foundation Trust, UK; b Warwick Clinical Trials Unit, University of Warwick, UK; c Welsh Ambulance Services NHS Trust, UK; d West Midlands Ambulance Service University NHS Foundation Trust, UK; e North East Ambulance Service NHS Foundation Trust, UK; f London Ambulance Service NHS Trust, UK

**Keywords:** Out-of-hospital Cardiac Arrest, Randomised Controlled Trial, Cardiopulmonary Resuscitation, Methodology, Trial design

## Abstract

**Background:**

PARAMEDIC2 was a medicines trial comparing adrenaline with placebo in out-of-hospital cardiac arrest (OHCA). At the time, United Kingdom (UK) Emergency Medical Systems (EMS) were inexperienced in delivering such research.

**Aim:**

To identify barriers and facilitators to delivery of the PARAMEDIC2 (Adrenaline) trial by five UK NHS EMS.

**Methods:**

This qualitative study took a grounded theory approach to thematic analysis of workshop data. Members of the trial teams from each service attended a workshop in November 2018 and discussed their experiences in answer to two prompt questions. Data were coded and themes presented.

**Results:**

Three main themes were identified: *professionalism, organisational investment* and *unique features of EMS*. The study provided an opportunity for recruiting paramedics and research paramedics to demonstrate their *professionalism*. Research paramedics felt it was part of their professional duty to initiate discussions with the patient/family regarding the trial rather than leave this task to the hospital teams as would usually happen. *Organisational investment* was reflected by prioritising trial training and further development of research paramedics. By these means, research culture was developed. The *unique features of EMS* such as geographical challenges were often addressed with technological solutions and through building relationships with internal teams.

**Conclusion:**

Barriers to trial delivery included infrequent exposure to the condition of interest and lack of continuity in research paramedic roles. Facilitators identified included flexibility of the research protocol, and organisational investment in the development of research paramedics.

Participating in PARAMEDIC2 was challenging for the EMS involved, but ultimately strengthened their research culture.

## Introduction

The last decade has seen the successful completion of multiple trials conducted in United Kingdom Emergency Medical Systems (EMS) in out-of-hospital cardiac arrest management and stroke treatments,^1^, ^2^, ^3^ with several more in progress or awaiting results.^4^, ^5^, ^6^ The prehospital environment is a complex research setting where the recruiting staff are usually not specialist research clinicians. Recruitment can happen in diverse and sometimes challenging locations and prior informed consent is not always possible.^7^ With healthcare budgets being squeezed ever tighter it is vital that prehospital trials are optimised for efficiency to meet time, target and budget. On average, UK trials cost just under £8000 per patient due to so many not reaching their target,^8^ whilst avoidably wasting around 85% of resources.^9^ It is clear learning should be disseminated about what does and does not work to help future trials succeed.^10^

The Prehospital Adrenaline for Cardiac Arrest (PACA) trial was conducted in Australia as a randomised control trial (RCT) comparing adrenaline with placebo in out-of-hospital cardiac arrest (OHCA).^11^ A lack of paramedic and public support led to a failure to achieve target recruitment but gave insights into training strategies and public awareness campaigns which informed the development of PARAMEDIC2.^12^

PARAMEDIC2 was an RCT investigating the safety and effectiveness of adrenaline versus placebo in OHCA.^12^ The trial was successfully delivered by five UK EMS between 2014 and 2018. Following trial closure, members of the research teams from the five participating EMS met to discuss their experiences of conducting the trial. This work aims to identify barriers and facilitators to the successful delivery of the PARAMEDIC2 (Adrenaline) trial in UK EMS.

## Methods

### Study design & setting

This study sought to understand the perceptions of those delivering the trial, via a grounded theory approach to thematic analysis and did so within an interpretivist paradigm. This assumes a socially constructed reality and allows open-ended questions to enable a broad exploration of the topic.^13^ A workshop was hosted by the Warwick Clinical Trials Unit (WCTU) (the trial-coordinating CTU) in November 2018 and attended by members of the trial teams involved in PARAMEDIC2 trial delivery from each participating EMS. Discussions took place over three hours, until no new topics were identified and data saturation was achieved. Workshop participants took turns to facilitate discussion on the various topics raised with the aim of avoiding privileging any one perspective. It was felt that due to the note-takers’ positions within the group as peers and colleagues, their familiarity with the topics raised would enable them to identify and capture the pertinent issues rather than needing to audio-record the workshop. A lack of audio-recording also encouraged participants to speak freely.^14^

### Participants

The views of those tasked with delivering the PARAMEDIC-2 trial in the five participating EMS in England and Wales were sought. Purposive sampling was used to identify ten potential workshop participants who were approached by email and invited to attend a one-day workshop. Up to two representatives from each of the participating EMS were invited, with the aim that a research manager and research paramedic from each service would participate. Managers played a key role in the project management and Research Paramedics in delivery of the trial. Specifically, Research Paramedics delivered training to Ambulance Paramedics, collected data from patient clinical records, sought consent from patients in hospital, monitored trial medicines and met with patients to conduct follow-up assessments. (Ambulance Paramedics undertook patient enrolment in this trial).

### Procedures

Two open ended prompt questions were used to initiate discussion:

1. If you were to do this trial again, what would you do the same/differently?

2. What are the key things that you would want to share with other researchers about undertaking this trial?

Topics were suggested by participants and then discussed in further detail. Notes were taken by HP and CS.

### Data analysis

A grounded theory approach was taken to data analysis with no *a priori* themes. Themes were identified via a process of thematic analysis.^15^ Firstly, field notes were collated and read through to increase familiarisation with the data set. Data were then coded manually by a single researcher (HP); following multiple coding sweeps, themes were generated. Emergent issues were then thematically grouped across the levels of coding (data extracts and whole data set) and then grouped into overarching themes until the phenomena of experiences of conducting the PARAMEDIC2 trial were explained.^16^ These were reviewed and agreed by all authors. Member checking was conducted by email and suggested changes agreed by majority consensus. Face validity was confirmed by all authors and all authors contributed to the final report.

## Ethics

Ethical approval was not required as this research involved NHS staff recruited as research participants by virtue of their professional role.

## Results

Seven participants attended the meeting comprised of three research paramedics, two Research and Development (R&D) Leads (one clinical, one non-clinical), one Clinical Director and a Trial Co-ordinator from WCTU. Two R&D Leads were absent from the workshop but later contributed via telephone and email. Contributors are listed in Table 1; 71% were female.

**Table 1:** Contributors.

Service 1	R&D* Lead x 1; Research Paramedic x 1
Service 2	Clinical Director x 1; Research Paramedic x 1
Service 3	R&D Lead x 1
Service 4	R&D Lead x 1; Research paramedic x 1
Service 5	R&D Lead x 1
WCTU^**^ staff	Trial Co-ordinator

*R&D = Research and Development.

**WCTU = Warwick Clinical Trials Unit.

**Fig. 1:**
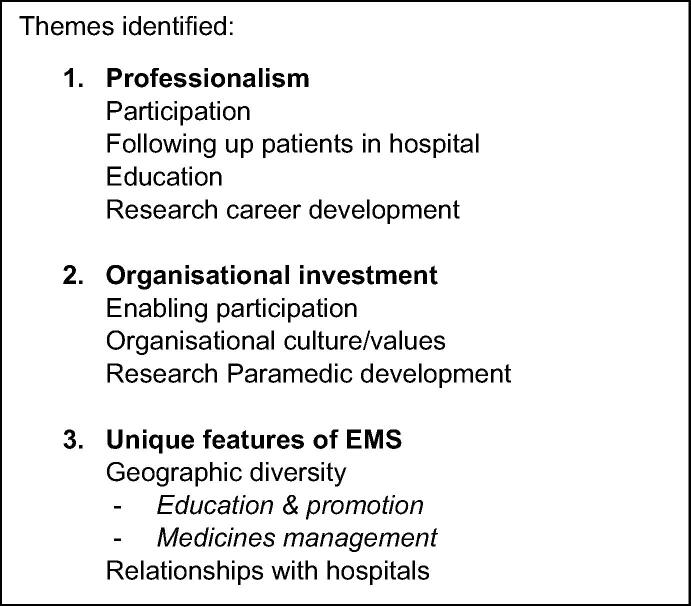
Themes and sub-themes identified.

The three main themes and sub-themes identified are shown in Fig. 1.

## Theme 1: Professionalism

### Participation

Research delivery teams in the workshop noted that ambulance paramedics saw trial involvement, either through patient recruitment or through support of that recruitment, as an opportunity to meet an aspect of their professional requirements.

Concerns were identified amongst some individuals regarding the ethics of recruiting patients under a waiver of consent model. Trial training usually addressed such concerns and the visibility of the trial in the media provided reassurance of public support. This was reinforced by the ability for members of the public to express a prior wish not to be enrolled by requesting and wearing a ‘no study’ bracelet. Such initiatives supported individual staff members’ decision to participate in the trial.

### Following up patients in hospital

Despite some research paramedics having long distances to travel to meet with patients in hospitals, all agreed that it should be the role of the EMS rather than the hospital teams to initiate discussions with the patient/family regarding the trial. It was noted that.

*“People want to have ‘soft conversations’ about what happened, not just ‘hard conversations’ about consent”.*

Research paramedics felt that it was part of their professional duty to have ownership of these conversations. Patients and their families usually had many questions about what happened on the day of their arrest and research paramedics were well placed to answer these given their experience of managing OHCA. This also provided some consistency as the same research paramedics conducted follow-up visits at three months.

### Education

The group agreed that the trial training delivered more than raised awareness of process and procedure. Completing the training prepared staff for research and signing up to take part in patient recruitment signified an embracing of evidence-based practice (EBP). It was noted that research education was:

*“more than just training staff on how to follow procedures. Also about preparing them for research in their everyday practice”*

Workshop participants noted that this was often as important for graduate paramedics as non-graduate paramedics since they had observed variability in research-related offering on undergraduate programmes.

The visibility and availability of research paramedics was essential in developing professional understanding of the trial. Whether through training delivery or crew-room conversations this was paramedics’ opportunity to challenge and have their concerns addressed.

### Research career development

For organisations where research is relatively new it is essential to build organisational memory to support continuity of activity. The research paramedic role seems less clearly defined than that of the more established hospital-based research nurse role. EMS tend not to delineate roles in terms of research delivery and research support. Their research teams tend to be very small and research paramedics are usually involved with all aspects of the research process. Whilst continuity is important for the organisation, this may be problematic for the individual paramedic wishing to develop research skills. Those employed full-time for this trial have felt disadvantaged in terms of missing out on clinical development opportunities. Workshop participants reported that the inherent pay cut resulting from moving off shift work had disincentivised many potentially good candidates from applying for research roles. However, offering a part-time/split role has provided opportunities for paramedics to extend their skill set without having to abandon their clinical practice.

## Theme 2: Organisational Investment

### Enabling participation

Organisational participation was facilitated by allowing adaptation of processes to local need. For example, the method by which local trial teams were notified of an enrolment varied between EMS. This flexibility enabled teams to use existing local solutions with known effectiveness rather than instigating potentially less effective new processes. For example, one service was notified and passed enrolment details via telephone call from ambulance paramedics to the ‘Clinical Desk’ which was available around the clock, whereas research paramedics tended to work office hours.

Individual participation was encouraged in different ways across organisations. Staff in two EMS were required to complete the trial training as part of their annual mandatory provision and could then choose whether to sign-up to take part. Paramedics in one EMS could opt to complete training during scheduled continuous professional development (CPD) time whilst the remaining two offered it for completion in staff’s own time. One of the latter EMS offered staff payment for training whilst the others did not. This flexibility was felt to be a facilitator as it meant EMS were not tied to a mode of delivery that might not suit their local situation. Whilst trial participation was not compulsory the different models of training offered suggested different expectations between the EMS regarding involvement.

Both individuals and organisations drew upon the authority of experts such as the British Heart Foundation and the Health and Social Care Professions Council. Their endorsement of the project supported and encouraged paramedic participation.

### Research paramedic development

The learning from this trial risks being lost from the ‘organisational memory’ of each EMS due to the unique way in which research posts are funded. Research paramedic posts are typically linked to individual trials. This has led to concerns regarding how the role fits into the professional career framework and various models are currently being implemented to support paramedics in their development. The experiential learning gained by research paramedics engaged in trials such as PARAMEDIC2 requires the underpinning academic development to have a meaningful impact on individuals’ career development. All authors agreed that funded support for individual development demonstrates the commitment of the organisation and reinforces the expectation that they will continue working in this area post-qualification. As an example, we noted that:

*“EMS X has funded masters’ scheme to support Advanced Practitioners; includes research module; research team also offering research masterclasses with external speakers to reinforce expectation they will continue to be involved (in research) post- qualifying”*

### Organisational culture/values

Workshop participants felt that where services included the trial in their mandatory training provision, this sent a clear message of the EMS’s commitment to research. It was recognised by workshop participants that time and financial constraints precluded some organisations from supporting face-to-face delivery of the training package. Other EMS demonstrated their commitment to research by fully or partially funding research modules for advanced practitioners.

Participating organisations anticipated and prepared for media enquiries regarding this potentially controversial trial. This was viewed positively as an opportunity to proactively communicate the values of the EMS as well as their support for the trial.

## Theme 3: Unique features of Emergency Medical Systems

### Geographic diversity

#### Education & promotion

A major challenge to delivering prehospital research is ensuring timely and consistent communication of information, training, and updates. Communication was a resource-intensive activity as so many stakeholders needed to be kept informed of the progress of the trial. All EMS valued the engagement of their Communications teams in this ongoing activity. Keeping the trial visible and fresh in people’s minds was a challenge. It was felt in one EMS that trial fatigue was appearing in the latter months of patient recruitment. This was evident in the occurrence of an increased number of errors suggesting that gradual closure of the trial, starting with those stations open the longest would be beneficial. This would need to be balanced against a longer whole-trial recruitment period.

Due to the infrequency of exposure of individual paramedics to the condition of interest, large numbers needed to be trained. Training was delivered to the mobile workforce via both eLearning and face-to-face sessions, with some mostly training by eLearning, and others predominantly via face-to-face delivery. Whilst eLearning was successful, some EMS reported that paramedics were becoming increasingly unenthusiastic about this method of delivery. It was commented that:

*“…whilst some (people) previously assumed eLearning could solve all our training problems, now finding that it can actually be quite disengaging”*

Most participants reported greater enthusiasm for face-to-face delivery of the training as this allowed concerns to be addressed in a timely fashion. However, eLearning was found to be a useful means of delivering refresher training. Maximising the visibility and availability of research paramedics was achieved by basing them at ambulance stations. Although sometimes challenging in terms of space and physical resources, this approach enabled conversations to continue throughout the duration of the trial.

Some EMS trained only recruiting paramedics; others trained all grades of frontline operational staff. The latter approach was felt to be a facilitator in services where it applied, since it increased awareness of the trial and minimised the chance of errors. It also promoted evidence-based practice more widely. Approaches to incentivising training varied with some services paying overtime, others providing food but most providing no incentive. The latter approach was taken when services delivered education to all staff members rather than just to recruiting paramedics; a consistent message to the whole team was prioritised over payments to staff which were prohibitively expensive. It was felt that small incentives provided throughout the trial, such as pens and chocolates for the crew room, had a greater impact than expensive launch events. Such events occurred, by definition, early in the life of the trial, leaving little resource for maintaining interest later in the trial when enthusiasm may have waned. Incentives were felt to be facilitators since it not only encouraged initial engagement, but also acted as a reminder of the trial.

#### Medicines management

Maximising availability of Investigational Medicinal Product (IMP) whilst maintaining a strict audit trail was challenging. A mobile workforce, only some of whom were trial-trained, together with the unpredictable movement of vehicles between base locations meant that constant and careful monitoring of trial pack locations was essential to prevent loss or inappropriate use. Ideally, IMP would have been personal issue but the financial implications precluded this approach.

A more pragmatic solution relied on paramedics signing trial packs in and out of the medicines store for each shift. This worked well in organisations where this was part of normal medicines management procedure. Where this was an ‘additional’ task, or if paramedics changed vehicles mid-shift, it was sometimes forgotten. This led to missed enrolment opportunities and the additional task of repatriating IMP. An alternative solution, appropriate where the whole organisation was involved, was to keep packs on vehicles and include them on the daily check-sheets used by the teams responsible for stocking the vehicles.

### Relationships with hospitals

EMS were reliant on hospital research teams to track patients’ progress through the hospital. The level of engagement of local collaborators and Principal Investigators varied enormously with some having little day-to-day involvement and others being instrumental in establishing and maintaining contacts.

Research paramedics obtained ‘letters of access’ enabling contact with trial patients. In most areas this worked well, but hospital teams appeared to struggle with the idea of paramedics coming onto the ward to seek consent as this was usually the role of the research nurses. Although higher-level decision-makers had allowed access, often research paramedics were not allowed access to hospital notes and had to write their notes on a separate piece of paper before giving to a member of staff to add to the patient’s notes. The problem diminished as ward staff became more accustomed to research paramedics’ visits.

Although most of the workload for the trial was undertaken by the EMS teams, the amount of work involved for the hospital teams in terms of tracking patients and finding notes was not inconsiderable. However, since consent was sought by research paramedics the hospitals did not receive direct financial recognition for this activity. But despite this, workshop participants did not feel this was a barrier to delivery of the research, citing instead goodwill and encouragement from the hospital research teams.

## Discussion

Several inherent key features were identified in the research design and setting that contributed to the success of the PARAMEDIC2 trial. The study provided an opportunity for paramedics to demonstrate their professionalism. The ambulance paramedics who recruited patients viewed their participation as an opportunity not only to answer the research question, but also to demonstrate their professional standards. The research paramedics involved in coordinating trial delivery felt that their experience working on the trial enriched their careers and valued the opportunity to promote evidence-based practice through training their frontline colleagues on the trial. Workshop participants felt that the research culture in their organisations was reflected by how each prioritised trial training and further development of research paramedics. The unique features of EMS presented geographical challenges which required significant investment of time and resource but were often addressed with technological solutions such as training via eLearning. Building relationships with internal teams such as pharmacy and external hospital teams was essential to the success of the project.

It was only in September 2023 that the HCPC introduced *the engagement of service users in research* as a professional standard.^17^ But, prior to this, some paramedics recognised the value of trial involvement as an opportunity to learn about research in preparation for the anticipated regulatory requirements to come. The PARAMEDIC2 trial was one of a small number of large trials that opened opportunities for paramedics to pursue research careers.^18^ The value of the research paramedic role was recognised by workshop participants, not only in research delivery and education but also in the development of organisational memory. Such memory is developed in both policies and the minds of individuals and enables a quicker response to similar future situations.^19^ Organisational memory can be established through building continuity within the research workforce which, in turn, supports development of a research culture within an EMS.^20^ However, it was evident that the short-term nature of most research posts threatened the development of such memory and culture. It is notable that some of the barriers to clinical academic careers noted in 2018 are still national issues for paramedics five years later.^17^ The development of research culture in an organisation requires not only advocacy from motivated research-literate individuals, but also action by receptive managers.^21^ Fully invested organisations demonstrated their commitment in different ways. Engagement with local/national media or inclusion of trial training on the annual mandatory provision were examples of how organisations demonstrated that they valued evidence-based practice which in turn supported development of a research culture.^21^.

Training large numbers of geographically disparate individuals was challenging. Although not ubiquitous, as it is now, at the time of this trial eLearning had already been found to be non-inferior to traditional training methods in developing knowledge in healthcare learners.^22^ In common with a contemporaneous UK prehospital study, large numbers of staff working in different locations were successfully trained using an online training package.^23^ Medicines management across a wide geography was also challenging, but the recommended strategy of involving a pharmacist in the design stage was hugely beneficial in anticipating sources of error and planning accordingly.^24^.

This study presents the collective views and experiences of those involved with delivery of PARAMEDIC2. A strength of this work is that all the participating EMS took part and so may be considered representative, but there were some limitations. Firstly, only a small number of participants from each service took part so alternative views and opinions may have been excluded. Secondly, the workshop was not audio recorded but minuted by two independent note-takers and reviewed by all contributors soon after the workshop. Perhaps the main limitation was that only the views of those running the trial at each site were sought and not those recruiting patients. However, their views have been captured and reported elsewhere.^25^, ^26^, ^27^.

The following recommendations are offered. Firstly, the Research Paramedic role needs permanency to give both individuals and their employing organisations continuity and job security. Secondly, clinical academic roles should be encouraged to maintain and strengthen links between operational and research functions. Future research into barriers and facilitators is warranted to promote even wider engagement of EMS in clinical research.

## Conclusions

Factors such as infrequent exposure to the condition of interest (OHCA), additional medicines tasks and lack of continuity in research paramedic roles were considered barriers to trial delivery. Facilitators to the success of the trial included building flexibility into the research protocol to allow local variation in trial delivery, EMS prioritising trial training and investing in the development of research paramedics.

The PARAMEDIC2 trial was the largest prehospital cardiac arrest medicines trial ever undertaken in Europe. Its delivery by UK EMS presented both challenges and opportunities for those involved which ultimately developed the research culture within those services.

## Funding

This research did not receive any specific grant from funding agencies in the public, commercial, or not-for-profit sectors. The PARAMEDIC2 trial was funded by the National Institute for Health Research HTA Programme (project number HTA – 12/127/126). All authors or their employers had been supported by this grant funding. HP holds a grant from the National Institute for Health and Social Care Research (NIHR)/Health Education England (ICA-CDRF-2018-04-ST2-05). The views expressed are those of the authors and not necessarily those of the NHS, the NIHR or the Department of Health.

## CRediT authorship contribution statement

**Helen Pocock:** Writing – review & editing, Writing – original draft, Methodology, Investigation, Formal analysis, Conceptualization. **Nigel Rees:** Writing – review & editing, Methodology, Formal analysis. **Imogen Gunson:** Writing – review & editing, Writing – original draft, Methodology, Formal analysis. **Mark Docherty:** Writing – review & editing, Formal analysis. **Karl Charlton:** Writing – review & editing, Writing – original draft, Methodology, Formal analysis. **Michelle Jackson:** Writing – review & editing, Methodology, Formal analysis. **Charlotte Scomparin:** Writing – review & editing, Methodology, Investigation, Formal analysis. **Ed England:** Writing – review & editing, Writing – original draft, Formal analysis. **Rachael Fothergill:** Writing – review & editing, Methodology, Formal analysis.

## Declaration of competing interest

The authors declare the following financial interests/personal relationships which may be considered as potential competing interests: HP is a member of the International Liaison Committee on Resuscitation (ILCOR) Advanced Life Support task force committee. She holds a grant from the National Institute for Health and Social Care Research (NIHR)/Health Education England (ICA-CDRF-2018-04-ST2-05). NR, IG, MD, KC, MJ, CS, EE, RF: Nil.
